# Domestic (re)infestation risk with the main vector *Triatoma infestans* increases with surrounding green vegetation and social vulnerability in the Argentine Chaco

**DOI:** 10.1186/s13071-024-06324-3

**Published:** 2024-05-27

**Authors:** Dario E. Elias, Marta V. Cardinal, Natalia P. Macchiaverna, Gustavo F. Enriquez, Ricardo E. Gürtler, M. Sol Gaspe

**Affiliations:** 1 Laboratorio de Eco-Epidemiología, Facultad de Ciencias Exactas y Naturales, Universidad de Buenos Aires, Ciudad Autónoma de Buenos Aires, Argentina; 2 Instituto de Ecología, Genética y Evolución de Buenos Aires (IEGEBA), Consejo Nacional de Investigaciones Científicas y Técnicas (CONICET)-Universidad de Buenos Aires, Ciudad Autónoma de Buenos Aires, Argentina

**Keywords:** Vector-borne diseases, Chagas, Social disparities, NDVI, Spatial analysis

## Abstract

**Background:**

Chagas disease, caused by *Trypanosoma cruzi*, is still a public health problem in Latin America and in the Southern Cone countries, where *Triatoma infestans* is the main vector. We evaluated the relationships among the density of green vegetation around rural houses, sociodemographic characteristics, and domestic (re)infestation with *T. infestans* while accounting for their spatial dependence in the municipality of Pampa del Indio between 2007 and 2016.

**Methods:**

The study comprised sociodemographic and ecological variables from 734 rural houses with no missing data. Green vegetation density surrounding houses was estimated by the normalized difference vegetation index (NDVI). We used a hierarchical Bayesian logistic regression composed of fixed effects and spatial random effects to estimate domestic infestation risk and quantile regressions to evaluate the association between surrounding NDVI and selected sociodemographic variables.

**Results:**

Qom ethnicity and the number of poultry were negatively associated with surrounding NDVI, whereas overcrowding was positively associated with surrounding NDVI. Hierarchical Bayesian models identified that domestic infestation was positively associated with surrounding NDVI, suitable walls for triatomines, and overcrowding over both intervention periods. Preintervention domestic infestation also was positively associated with Qom ethnicity. Models with spatial random effects performed better than models without spatial effects. The former identified geographic areas with a domestic infestation risk not accounted for by fixed-effect variables.

**Conclusions:**

Domestic infestation with *T. infestans* was associated with the density of green vegetation surrounding rural houses and social vulnerability over a decade of sustained vector control interventions. High density of green vegetation surrounding rural houses was associated with households with more vulnerable social conditions. Evaluation of domestic infestation risk should simultaneously consider social, landscape and spatial effects to control for their mutual dependency. Hierarchical Bayesian models provided a proficient methodology to identify areas for targeted triatomine and disease surveillance and control.

**Graphical Abstract:**

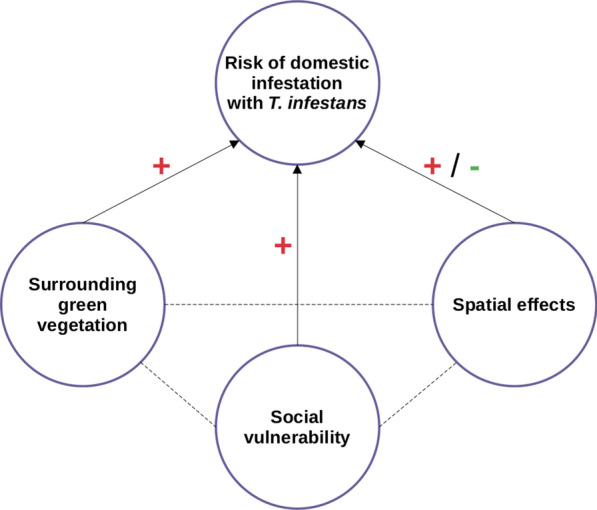

**Supplementary Information:**

The online version contains supplementary material available at 10.1186/s13071-024-06324-3.

## Background

Chagas disease, caused by *Trypanosoma cruzi*, is a neglected tropical disease and one of the major vector-borne diseases in Latin America, affecting 6 million people [1]. *Triatoma infestans* is the main vector of *T. cruzi* in the Southern Cone countries and has been the target of an insecticide-based regional elimination program that interrupted vectorial transmission to humans in various countries [2, 3]. However, in the Gran Chaco ecoregion, domestic vector-borne transmission of *T. cruzi* still occurs, albeit with lower incidence levels than 20 years ago [4].

Domestic infestation with triatomines is strongly associated with ecological and sociodemographic features. Ecological risk factors related to infestation have extensively been explored, whereas social aspects have been studied to a lesser extent [5]. Residential overcrowding, household educational level, wall building materials and indoor presence of poultry and dogs were main determinants of house infestation in different settings [6–9]. These factors lay in the intersection between the social and ecological domains and may be considered proxies of social disparities.

The transmission of *T. cruzi* encompasses widely heterogeneous eco-epidemiological scenarios across the Americas, from sylvatic triatomine species invading domiciles to highly domiciliated species [10, 11]. Therefore, the association between house infestation and the surrounding environment has yielded different results. For domiciliated vectors, such as *T. infestans* in Argentina, Cardozo et al. [12] showed that house invasion by triatomines was more frequent in isolated houses located near forest fragments within disturbed landscapes. Weinberg et al. [13] showed that clusters of houses with high domestic infestation with *T. infestans* had greater forest coverage and smaller cultivated areas than zones with low domestic infestation. The normalized difference vegetation index (NDVI), which is widely used to estimate green vegetation density based on satellite images [14], was positively associated with domestic or house infestation with *T. infestans* at the department and country levels [15–17]. Different causal pathways have been hypothesized regarding the association between the surrounding vegetation of houses and domestic infestation, such as that the surrounding vegetation could offer greater availability of refuges to triatomines or that the occurrence of more dispersed houses could favor the attraction of dispersing triatomines by artificial lights [12, 18]. However, social characteristics could play a relevant role in the association between the risk of infestation and environmental characteristics; for example, the risk attributed to living isolated in the vicinity of forest fragments could also be related to the social vulnerability in which marginalized families live.

Few studies integrated NDVI, as a surrogate of vegetation cover, and sociodemographic data in relation to house infestation with triatomines. King et al. (2011) [19] reported domestic infestation with *Triatoma dimidiata* was associated with surrounding mean NDVI and house walls of palm and straw in Jutiapa, Guatemala. By contrast, Brito et al. [20] found a negative association between house infestation with *Rhodnius neglectus* and NDVI values adjusted for municipality-level socioeconomic indices in Tocantins, Brazil. Gonçalves et al. [21] revealed that NDVI values adjusted for sociodemographic characteristics at the census tract level were positively related to the occurrence of triatomines in northern Minas Gerais, Brazil. However, the environmental scenarios and the ecological features of the main triatomine vector in the Argentine Chaco, mainly its degree of domiciliation and the virtual absence of sylvatic foci, are different from those addressed in the aforementioned studies. Therefore, the causal and non-causal hypotheses of the association among green vegetation, sociodemographic characteristics and triatomine infestation in this region would probably follow different pathways that deserve further research. To our knowledge, no study has simultaneously analyzed NDVI surrounding houses and sociodemographic data at the household level involving *T. infestans* throughout its distribution range.

This study is part of an ongoing research and control program on the eco-epidemiology of Chagas disease in the municipality of Pampa del Indio (Chaco province, Argentina) initiated in 2007. A series of district-wide interventions achieved the quasi-elimination of *T. infestans* [22] and the virtual interruption of vector-borne transmission of *T. cruzi* at the municipality-wide level [23, 24]. House infestation in operational areas within Pampa del Indio was associated with residential overcrowding, household educational level, Qom ethnicity, domestic insecticide use and mud walls [7, 25, 26]. In the current study, using a hierarchical Bayesian framework that allows for the spatial features of the processes involved, we modeled the relationship among the occurrence of domestic (re)infestation with *T. infestans*, green vegetation density surrounding rural houses (estimated by NDVI) and selected household sociodemographic characteristics over a 10-year period. A key aspect of this study is the inclusion of the spatial component, as several studies have rejected the hypothesis of spatial independence of house infestation with triatomines [16, 27, 28]. Based on empirical evidence collected in northern Argentina [7, 17], we hypothesized that disadvantageous sociodemographic characteristics (e.g. overcrowding) and higher NDVI surrounding houses were directly associated with a higher risk of domestic infestation with *T. infestans*. Second, we hypothesized that the surrounding NDVI of houses was associated with household sociodemographic characteristics.

## Methods

### Study area

The Gran Chaco ecoregion comprises two regions: the humid Chaco, composed of tropical grasslands, savannas and shrublands, and the dry Chaco, a semi-arid region of dry forests and savannas [29].

This study was carried out in the municipality of Pampa del Indio, Chaco province, Argentina, located in the transition between the humid and dry Chaco. The municipality extends over approximately 60 km by 30 km and includes two loosely connected urban centers (Pampa del Indio and Pueblo Viejo), a peri-urban section (Parque Industrial) and 32 dispersed rural settlements. The rural section of the municipality was divided into four areas (I, II, II and IV) for operational purposes [22]. Based on a supervised classification, Rodríguez-Planes [30] showed that in Pampa del Indio the estimated percentage of land covered by different types of forests (with different degrees of degradation) was 74.4% and 72.4% in December 2006 and August 2015, respectively. The forests in the area have suffered a process of extraction and replacement that has reduced cover and increased contact between forest habitats and other land covers. Forest cover is deeply intertwined with other land covers [30]. In rural areas, the main productive activity was a subsistence economy, without extensive agriculture and with extensive livestock grazing mainly in the forests [30].

According to the 2010 national census, Pampa del Indio was inhabited by 15,287 people in 3862 housing units. The district is inhabited by two main groups: Qom, a formerly nomadic indigenous people organized in sedentary communities [31], and Creoles of European descent who arrived in the early 1910s. Chaco is among the provinces with the greatest social deprivation indices in Argentina [32].

### Study design and household surveys

The intervention program was designed to assess the effects of community-wide spraying with pyrethroid insecticide on house (re)infestation with *T. infestans* in a context of persistently infested neighboring districts [22]. The program covered two overlapping periods: preintervention (2007–2010) and postintervention (2008–2016). Surveys were conducted before and after community-wide spraying to assess house infestation with triatomines, collect sociodemographic data and decide whether a house was to be re-sprayed with insecticides.

House infestation with *T. infestans* was assessed by timed manual searches assisted with an aerosol to dislodge the insects (0.2% tetramethrin). Searches were conducted by personnel of the National or Provincial Chagas Program, supervised by one member of the research team. A house was considered infested if any live nymph or adult was found (excluding eggs). House infestation prevalence was 26.8% (range: 14.4–41.4%) in the preintervention period; it was rare (range: 1.9–3.7%) over 2–6 years postintervention and dropped to 0.7% (95% confidence interval, 0.28–1.29%) at endpoint across rural areas [22].

A household was defined as all the people who occupied a housing unit, including related and unrelated family members. A house was defined as a set of constructions that typically included a domestic habitat (or domicile, i.e. an independent structure used as human sleeping quarters) and peridomestic structures such as chicken coops, storerooms and corrals [7, 25]. In total, 2280 houses were registered between 2007 and 2016 and their location georeferenced with a GPS receiver (Garmin Legend; Garmin Ltd., Schaffhausen, Switzerland).

### Data preparation

#### Outcome and sociodemographic variables

We included selected sociodemographic variables of interest that had been surveyed across the four operational areas and had fewer missing data: self-reported ethnicity (Qom and Creole), overcrowding (number of people per sleeping quarter), presence of suitable walls for triatomines, number of poultry (mainly chickens), number of dogs and cats and presence of peridomestic structures. After excluding houses with missing data on the selected variables, the study base comprised 734 houses, which represents 32.7% of the houses ever surveyed across the follow-up (Additional file 1: Dataset S1). In the preintervention period, 91.2% of the houses in the subset were evaluated for triatomine infestation between 2007 and 2008, while in the postintervention period, 96.4% of the houses were evaluated between 2009 and 2016; thus, the fraction of houses that shared an overlapping time frame was minimal. In this study we focused on domestic infestation with *T. infestans* as the main outcome variable because domestic infestation has direct effects on vector-borne transmission risk of *T. cruzi* to humans [24]. As 15.1% of the houses in the subset did not have peridomestic structures, we did not pursue a similar analysis for peridomestic infestation.

#### Surrounding green vegetation

To estimate the green vegetation surrounding each house, we calculated the NDVI based on Landsat 7 ETM + Level 1 images downloaded from the EarthExplorer of the United States Geological Survey (USGS) [33]. The spatial resolution of these images was 30 m per pixel. They were gap filled and corrected atmospherically using the dark pixel method [34]. The NDVI was calculated from the spectral difference between red and infrared bands. The NDVI values oscillate between – 1 and 1; the higher the value, the greater the density of green vegetation, with values < 0 indicating absence of vegetation [14]. In the humid Chaco ecoregion, Bigerna et al. [35] showed that NDVI values close to 0.8 were associated with forests, such as riparian forests, whereas values around 0.6 were associated with the presence of dense or open savanna and flooding grassland.

For each year between 2007 and 2016, three satellite images with < 20% cloud coverage were selected for the period between November and April of the following year, when most of the triatomine surveys were carried out. After excluding images with cloud agglomerations in the study area, only three images could be selected for the preintervention period. Therefore, for each postintervention year, the same number of images were selected, which represents more than half of the median number of images available per year, prioritizing those with less cloud coverage and seeking to have similar amounts of images for each bimester. In the preintervention period, two images from November–December and one from March–April were selected, while in the postintervention period, a total of nine, seven and eight images for November–December, January–February and March–April were selected, respectively. The median value of each pixel was calculated for each intervention period. The surrounding NDVI of each house was calculated as the median NDVI considering a 1000-m radius buffer centered on the corresponding domicile. For these purposes, we used QGIS software [36] and R packages RStoolbox, sf and raster [37–39].

### Data analysis

#### Global spatial analyses

We used random labeling to test the null hypothesis of random occurrence of marks (i.e. domestic infestation status, surrounding NDVI and sociodemographic variables) among the fixed spatial distribution of all selected study houses for each intervention period. For qualitative marks, we used the L(r) Ripley statistic by the distance (r), and for quantitative marks we used the rho(r) mark variogram, which indicates whether neighboring households present similar mark values evaluated at each distance r [40]; lower rho values reflect more similar values. The 95% confidence envelope was obtained from 9999 Monte Carlo simulations using the R package spatstat [41].

#### Association between surrounding NDVI and sociodemographic variables

We used quantile regressions to estimate the association between the surrounding NDVI and household sociodemographic characteristics, since the distribution of surrounding NDVI did not fit a normal distribution (Shapiro-Wilk test, *W* = 0.99, *P* < 0.0001). While least-squares linear regression estimates the conditional mean of the response variable across values of the explanatory variables, quantile regression estimates the conditional quantiles of the response variable [42]. Quantile regressions were performed between the 0.1 and 0.9 quantiles of the surrounding NDVI distribution, with a step of 0.1. Overcrowding, number of poultry and number of dogs and cats were standardized. We included the intervention periods (pre- and postintervention) and areas (I, II, II and IV) as covariates and used R package quantreg [43].

#### Domestic infestation model

To estimate domestic infestation risks, we initially used a logistic regression model within a hierarchical Bayesian framework including fixed effects and spatial random effects. Consider y(i) a variable that indicates domestic infestation status (1: infested, 0: not infested) of a house at position i, with probability P(i) of being infested depending on house covariates and the risks posed by nearby houses. We assumed that y(i) follows a Bernoulli distribution:

$$y(i)|P(i)\sim Bernoulli\,\left( {P(i)} \right)$$

We used the logit link function:

$$logit\,\left( {P(i)} \right) = x(i)\upbeta +s(i)$$

where *x* is a vector of fixed-effect variables (sociodemographic and surrounding NDVI variables), β is a vector of fixed-effect coefficients, and *s* is the spatial random effect that follows a zero-mean Gaussian process with Matérn covariance function [44]. This covariance function is defined by two parameters: σ, which denotes the standard deviation, and ρ, which represents the distance where the spatial correlation between two points reaches 0.1. We used default priors for β, while for the ρ and σ we used the penalized complexity prior to induce tail probabilities of Pr (*ρ* < 100 m) = 0.05 and Pr (*σ* > 5) = 0.05 [45]. Overcrowding, number of poultry, number of dogs and cats and surrounding NDVI were standardized. Parameter estimation was done using an integrated nested Laplace approximation with the stochastic partial differential equation (SPDE) representation for the spatial effects, available from the R-INLA package [46, 47]. The SPDE approach represents a continuously indexed Gaussian field with Matérn covariance as a discretely indexed Gaussian Markov random field [48]. For this purpose, a representation of the base function defined in a triangulation of the domain is used. Based on suggestions by Righetto et al. [49], we created a constrained refined Delaunay triangulation with maximal edge length of 500 m and 2000 m for the inner domain (study region) and outer domain (outside the study area, to avoid border effects), respectively, and 20 m as the shortest allowed distance between points. We selected a maximal edge length of 500 m for the inner domain because it was the shortest distance for which all the models converged; it represents < 1/10 of the minimum range of the study area, and the ρ estimated by the models was at least seven times greater than it. We selected the shortest allowed distance between points as 20 m because it represented < 1/5 of the maximal edge length for the inner domain and was < the 10th percentile of the nearest neighbor distance distribution in the study datasets.

We ran the models for each separate intervention period to estimate the coefficients for the fixed effects and spatial random effects. To evaluate whether the inclusion of spatial random effects improved the fit, we ran the models with the fixed effects only and selected the most parsimonious model based on the minimization of the Watanabe-Akaike information criteria (WAIC) [50].

We analyzed the sensitivity of model results to variation in the radius used to estimate the surrounding NDVI by testing alternative radii (100, 500, 1500 and 2000 m).

We used the same methodology to estimate domestic infestation risk with the bigger dataset that included 86.9% (1982) of the surveyed houses with no missing values for ethnicity to validate the spatial effects model with a substantially larger dataset (Additional file 1: Dataset S2; Additional file 2: Text S1).

## Results

The percentage of houses with a domestic infestation with *T. infestans* was higher in the preintervention period (17.1%, *n* = 703) than in the postintervention period (5.2%, *n* = 734); (*χ*^2^: 50.7, df: 1, *P* < 0.0001). In univariate analyses, preintervention domestic infestation was positively associated with Qom ethnicity, suitable walls for triatomines and overcrowding, whereas postintervention domestic infestation was positively associated with an increase in surrounding NDVI (Table 1).

**Table 1:** Distribution of variables by intervention period and domestic infestation status

Period	Variable^a^	Total	Infested houses	Non-infested houses	Statistic	*P* value
Preintervention	Number of inspected houses	703	120	583	–	–
	Qom ethnicity	407 (57.9)	84 (70.0)	323 (55.4)	8.1	0.0044*
	Suitable walls for triatomines	406 (57.8)	93 (77.5)	313 (53.7)	22.2	< 0.0001*
	Presence of peridomestic structures	606 (86.2)	99 (82.5)	507 (87.0)	1.3	0.2518
	Overcrowding	2.5 (1.3, 4.0)	4.0 (2.0, 5.0)	2.0 (1.3, 3.9)	23455.0	< 0.0001*
	Number of poultry	15.0 (3.0, 30.0)	15.0 (5.0, 28.2)	15.0 (2.0, 30.0)	34786.5	0.9236
	Number of dogs and cats	3.0 (1.0, 5.0)	3.0 (1.0, 5.0)	3.0 (1.0, 5.0)	33853.0	0.5744
	Surrounding NDVI	0.6 (0.5, 0.6)	0.6 (0.6, 0.6)	0.6 (0.5, 0.6)	31643.0	0.0996
Postintervention	Number of inspected houses	734	38	696	–	–
	Qom ethnicity	421 (57.4)	21 (55.3)	400 (57.5)	0.0	0.9207
	Suitable walls for triatomines	420 (57.2)	27 (71.1)	393 (56.5)	2.6	0.1093
	Presence of peridomestic structures	624 (85.0)	34 (89.5)	590 (84.8)	0.3	0.5771
	Overcrowding	2.5 (1.3, 4.0)	3.4 (1.6, 4.8)	2.5 (1.3, 4.0)	11330.0	0.1354
	Number of poultry	15.0 (2.0, 30.0)	16.5 (10.0, 30.0)	15.0 (2.0, 30.0)	12322.0	0.4759
	Number of dogs and cats	3.0 (1.0, 5.0)	4.5 (1.0, 6.0)	3.0 (1.0, 5.0)	11492.0	0.1694
	Surrounding NDVI	0.6 (0.6, 0.6)	0.6 (0.6, 0.7)	0.6 (0.6, 0.6)	9853.0	0.0081*

^a^In Qom ethnicity, suitable walls for triatomines and presence of peridomestic structures variables, the quantity and percentage in parentheses are shown in Total, Infested houses and Non-infested houses columns; values in Statistic and *P* value columns correspond to the Chi-square test with df = 1. In overcrowding, number of poultry, number of dogs and cats and surrounding NDVI variables, the median and 25th and 75th percentiles in parentheses are shown in Total, Infested houses and Non-infested houses columns; values in Statistic and *P* value columns correspond to the Wilcoxon rank sum test. *NDVI* normalized difference vegetation index

^*^Statistically significant

Preintervention domestic infestation was spatially aggregated between 200 and 2820 m (Additional file 3: Figure S1). Over this range, Qom ethnicity, suitable walls for triatomines and surrounding NDVI were substantially aggregated in both intervention periods, and the presence of peridomestic structures showed significant spatial repulsion (Additional file 3: Figures S2–S5). Random global spatial patterns were observed for overcrowding, number of poultry and number of dogs and cats (Additional file 3: Figures S6–S8). Postintervention domestic infestation was not spatially aggregated (Additional file 3: Figure S1).

**Fig. 1:**
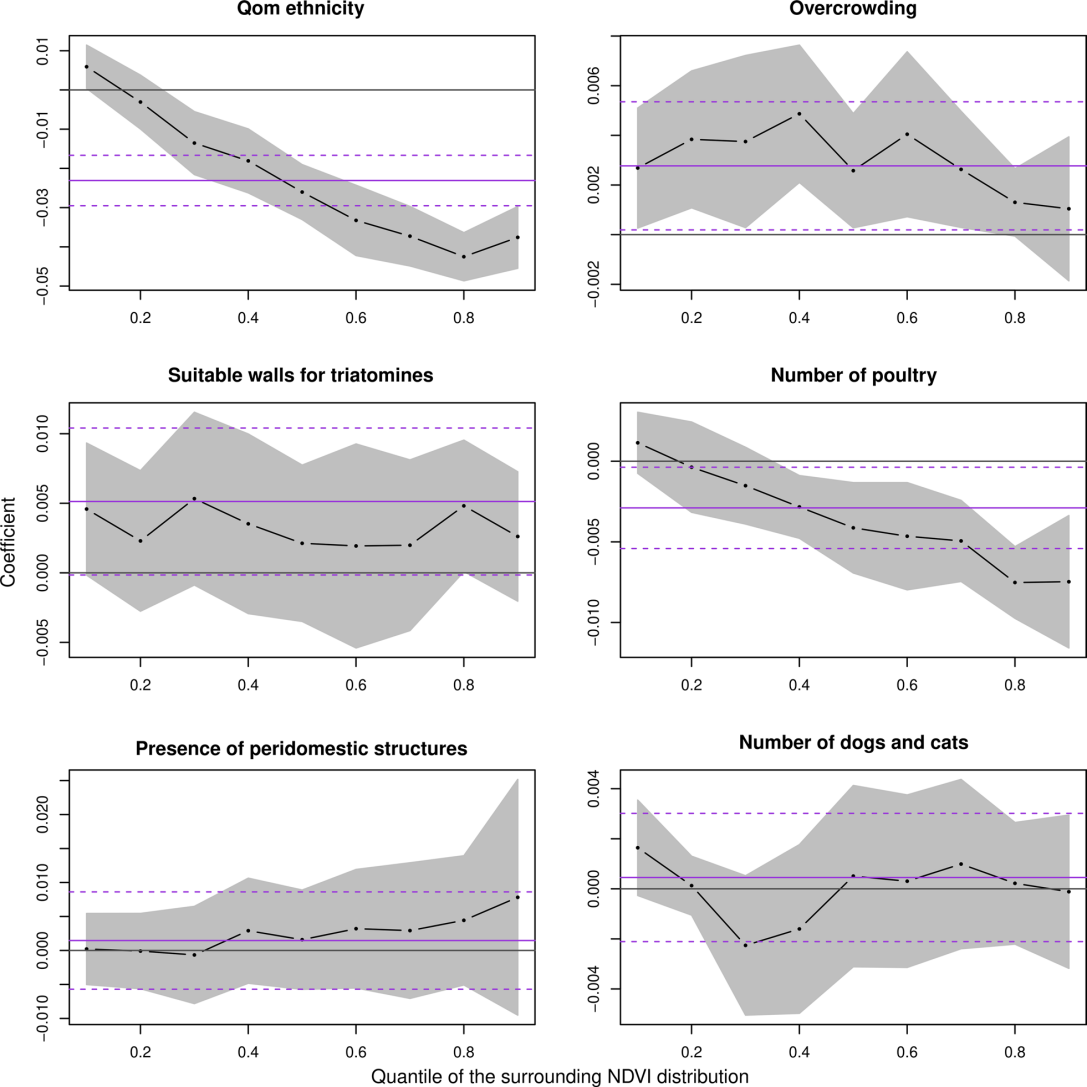
Association between surrounding NDVI and sociodemographic variables. The coefficients shown correspond to quantile regressions considering the surrounding NDVI as the outcome variable. Operational areas and the intervention period were included as covariates. The gray-shaded region indicates the 95% confidence interval of quantile regression coefficient. The violet solid line indicates the coefficient corresponding to an ordinary linear regression and the violet dotted lines its 95% confidence interval. *NDVI* normalized difference vegetation index

Using quantile regressions, Qom ethnicity and the household number of poultry were negatively associated with surrounding NDVI values in quantile ranges [0.3–0.9] and [0.4–0.9], respectively (Fig. 1, Additional file 4: Table S1). In contrast, overcrowding was positively associated with surrounding NDVI values between the 0.1 and 0.7 quantiles, and the presence of suitable walls for triatomines in the domicile showed a positive trend with surrounding NDVI across all quantiles (Fig. 1, Additional file 4: Table S1). Neither the presence of peridomestic structures nor the number of dogs and cats was significantly associated with surrounding NDVI.

**Fig. 2:**
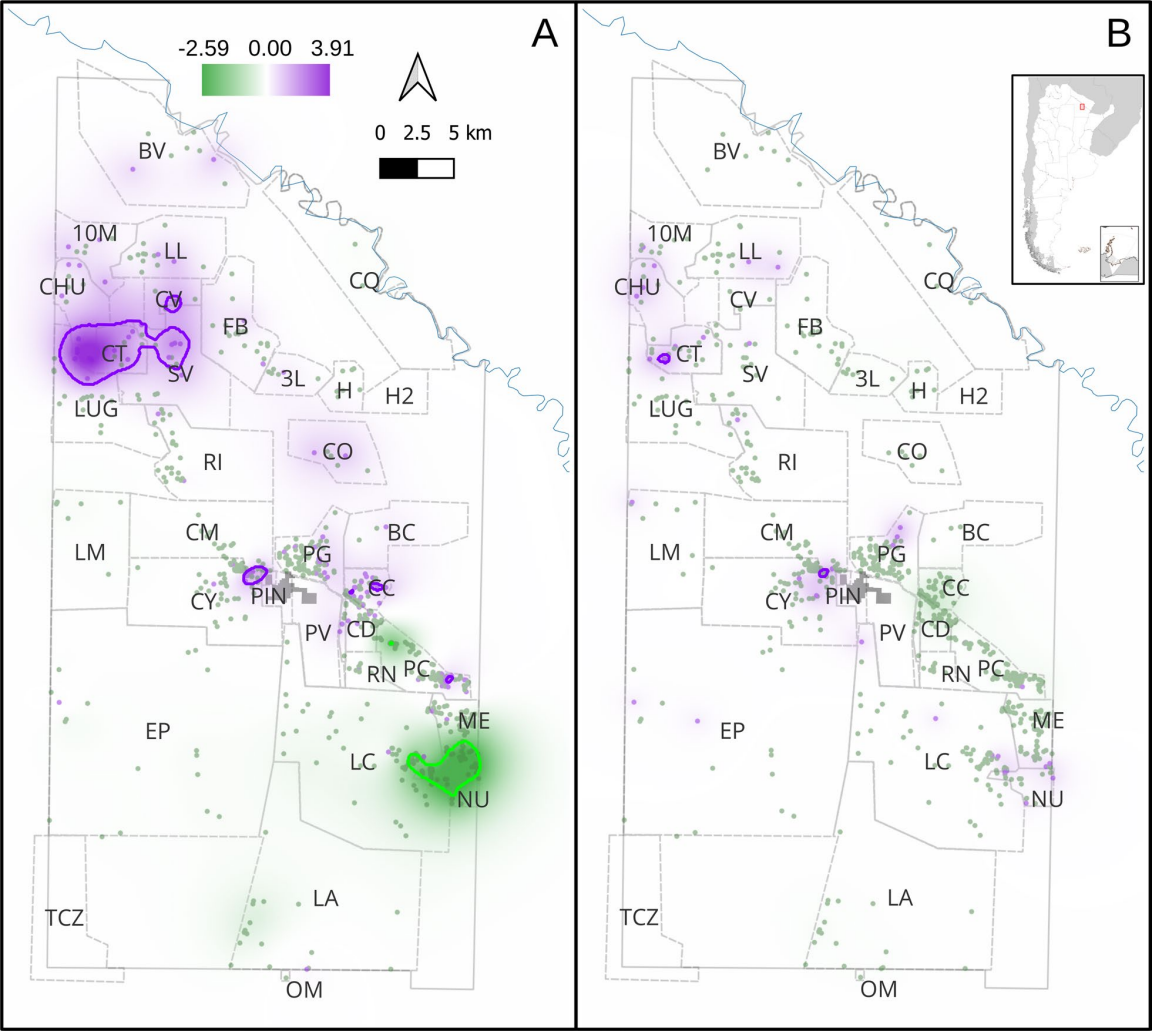
Observed domestic infestation and posterior median of spatial effect by intervention period. **A** preintervention and **B** postintervention. The regions delimited by violet and green lines correspond to those where the spatial effect did not include the value 0 in its 95% credible interval; in violet, spatial effects > 0; in green, effects < 0. The dots represent the houses (violet: infested, green: non-infested). The solid gray lines represent the municipality boundaries, the solid blue line represents the Bermejo River, and the dashed gray lines represent village boundaries. The gray region represents the urban conglomerate of Pampa del Indio. Village acronyms: *10 M* 10 de Mayo, *CT* Campo Los Toros, *CO* Colonia Ombú, *SV* El Salvaje, *CV* Los Ciervos, *FB* Fortín Brown, *H* La Herradura, *LL* La Loma, *BV* Las Bravas, *CHU* Las Chuñas, *RI* Santa Rita, *LUG* Santos Lugares, *3L* Tres Lagunas, *LC* Lote Cuatro, *NU* Campo Nuevo, *ME* Campo Medina, *LA* Cancha Larga, *OM* Pampa Ombú, *BC* La Barrancosa, *PG* Pampa Grande, *PC* Pampa Chica, *CC* Cuarta Legua Catorce, *CD* Cuarta Legua Diecisiete, *PV* Pueblo Viejo rural, *RN* El Rincón, *CQ* Campo Cacique, *H2* La Herradura 2, *CY* Campo Alemany, *CM* Colonia Mixta, *LM* Las Muñecas, *EP* ex-Parque, *TCZ* Tacuruzal, *PIN* Parque Industrial

Regarding domestic infestation risk, in both intervention periods the models including spatial random effects had lower WAIC values than the models without spatial random effects (511.5 vs. 609.7 in the preintervention period and 278.8 vs. 299.7 in the postintervention period). In the preintervention period, domestic infestation risk increased in households with Qom descent, overcrowded, having suitable walls for triatomines and with increasing surrounding NDVI. In the postintervention period, overcrowding, suitable walls and surrounding NDVI were associated with a higher risk of domestic infestation (Table 2). Regarding spatial effects, in the preintervention period, we identified eight geographic regions, ranging from 0.03 to 26.22 km^2^, where the spatial effect did not include 0 in its 95% credible interval (CI) (i.e. regions with a positive or negative risk of domestic infestation not explained by the fixed effect variables included in the models) (Fig. 2A). In particular, two large-sized regions were identified: one covering 26.22 km^2^ in the northwest (overlapping with Campo Los Toros, El Salvaje, Las Chuñas, Santos Lugares and Los Ciervos villages), associated with a higher domestic infestation risk. The second region, covering 10.19 km^2^ in the southeast section of the district (overlapping with Campo Nuevo, Lote Cuatro and Campo Medina villages), was associated with a lower domestic infestation risk. In the postintervention period, two geographic regions were associated with a higher risk of domestic infestation: one (0.34 km^2^) overlapped with Campo Los Toros village; the second region (0.24 km^2^) overlapped with Campo Alemany and Colonia Mixta villages (Fig. 2B).

**Table 2:** Coefficients of fixed and random effects of domestic infestation models

Variable	Preintervention^a^	Postintervention^a^
Intercept	−3.81 (−5.14 to −2.65)^b^	−4.76 (−6.62 to −3.18)^b^
Qom ethnicity	1.25 (0.43–2.12)^b^	0.14 (−0.88 to 1.22)
Overcrowding	0.56 (0.32–0.81)^b^	0.36 (0.01–0.72)^b^
Suitable walls for triatomines	0.97 (0.38–1.57)^b^	0.88 (0.06–1.71)^b^
Number of poultry	0.02 (−0.29 to 0.32)	0.04 (−0.36 to 0.44)
Number of dogs and cats	0.13 (−0.13 to 0.39)	0.12 (−0.25 to 0.49)
Surrounding NDVI	0.49 (0.04–0.97)^b^	0.56 (0.06–1.10)^b^
Presence of peridomestic structures	0.05 (−0.63 to 0.73)	0.44 (−0.78 to 1.66)
Range (ρ)	6716.71 (3746.86–15180.53)	3558.59 (735.58–18883.92)
Standard deviation (σ)	1.79 (1.25–2.66)	1.45 (1.04–2.06)

^a^Median and 95% credibility interval of coefficients are shown. *NDVI* normalized difference vegetation index

^b^Fixed effects that did not include 0 in their 95% credibility interval

We obtained no significant effects of surrounding NDVI on domestic infestation risk included in the models described above when other radial distances (100, 500, 1500 and 2000 m) were used to estimate the surrounding NDVIs in both intervention periods (Additional file 4: Table S2), except for the 500 m radius in the preintervention period (0.42, 95% CI 0.06–0.80). This coefficient did not differ significantly from that obtained with the 1000 m radius. With a radius of 1500 m, the coefficient showed a positive trend in the postintervention period (0.53, 95% CI −0.03 to 1.14). In addition, the WAIC values of the models with other radii were higher than those of the models with the 1000 m radius or exhibited a trivial difference (< 1) (Additional file 4: Table S2).

Nearly the same main outcomes were obtained with the bigger dataset including 1982 houses. The 95% CI of Qom ethnicity and surrounding NDVI for the subset of 734 study houses overlapped with those obtained with 1982 houses (Additional file 2: Table S3 and Figure S9). Likewise, in the analyses with both datasets, the largest-sized regions where the 95% CI for spatial effects did not include 0 also overlapped.

## Discussion

We found that overcrowding was associated with increasing values of surrounding NDVI, whereas Qom ethnicity and the number of poultry were associated with decreasing values of surrounding NDVI in rural houses of Pampa del Indio. Hierarchical Bayesian models integrated sociodemographic, environmental and spatial aspects and revealed that domestic infestation risk with *T. infestans* was associated with both the density of green vegetation surrounding the houses and sociodemographic factors across both intervention periods. In the preintervention period, surrounding NDVI, overcrowding, suitable walls for triatomines and Qom ethnicity were associated with a higher domestic infestation risk. Three of these variables (surrounding NDVI, overcrowding and suitable walls) were also associated with domestic infestation risk in the postintervention period. In both periods, we identified areas with a differential infestation risk not explained by the sociodemographic and environmental variables included in the models.

The well-known direct association between domestic infestation risk, overcrowding and suitable walls for triatomines [6, 8] reveals the influence of social disparities on the occurrence and persistence of house infestation, likewise in Area III of Pampa del Indio [7, 9]. Wall conditions are directly associated with the availability of appropriate refuges and triatomine abundance [51–53]. A plausible interpretation of the domestic infestation risk associated with overcrowding is that more human occupants per room (i.e. human density) increase the availability of blood meal sources, human-feeding success and the nutritional status of *T. infestans* populations [54, 55]. Host-feeding success is also affected by the relative contribution of other domestic bloodmeal sources, mainly chickens and dogs in the Chaco ecoregion [56], which can be of magnitude and correlates positively with the number of human residents. Although the household numbers of poultry, dogs and cats were not associated with domestic infestation risk, these variables did not consider whether these domestic hosts rested indoors or not. Overcrowding and wall conditions suitable for triatomines reflect social vulnerability and households that have less access to resources for prevention (e.g. insecticide use and improvement of house construction) along with disadvantageous structural conditions [7, 9].

Our results showed a positive association between surrounding green vegetation and domestic infestation risk, in agreement with some studies in different settings [17, 18]. High vegetation cover may be linked to a greater availability of refuges and hosts for sylvatic triatomine species, depending on the epidemiological scenario [18–21]. In the context of vector control interventions, house spraying with insecticides may trigger triatomine dispersal from the infested house to sylvatic or extra-peridomestic habitats and maintain a latent risk of house (re)infestation from these sources [57], especially where *T. infestans* has numerous sylvatic foci as in Bolivia [58]. In Pampa del Indio, sylvatic *Triatoma sordida* and *Panstrongylus* sp. were frequently collected but no sylvatic focus of *T. infestans* has been found so far [59, 60]. In the Dry Chaco ecoregion, sylvatic foci of *T. infestans* were detected between 110 and 2300 m from the nearest house after community-wide spraying with pyrethroids [57]; nearly all of them were associated with trees at ground level, fallen trees and tree stumps. Coincidentally, in the current study, although the highest infestation risk of surrounding green vegetation was observed within a 1000 m radius, a similar trend occurred within 500 and 1500 m radii. These distances fall within the estimated flight range of *T. infestans*, roughly 1.5 km [61–63]. In Area I of Pampa del Indio, a longitudinal study revealed a positive association between house infestation with *T. infestans* and high coverage of dry forest within 500 m of the house across both intervention periods, whereas postintervention house infestation was negatively associated with humid forests [30]. These analyses excluding sociodemographic characteristics corroborated our findings.

NDVI was associated with overcrowding (positively), the household number of poultry and Qom ethnicity (negatively); these relationships are likely rooted in socioeconomic conditions. In Pampa del Indio, rural households with more vulnerable social conditions had fewer livestock (including poultry) and therefore a lower number of peridomestic structures and smaller peridomestic areas; hence, surrounding NDVI could reach higher values. The negative association between surrounding NDVI and Qom ethnicity may be partially related to the current spatial organization of Qom households in Pampa del Indio, with houses heavily clustered in well-defined sections and within short distances from each other, a spatial pattern partly linked to collective land ownership [7]. The association between surrounding NDVI and sociodemographic characteristics hints at the social dimension underlying variation in NDVI and supports the need to simultaneously include both social and landscape characteristics in the assessment of domestic infestation risks to account for potential collinearity.

Environmental (e.g. proximity and conservation status of nearby forest fragments) and social drivers (isolation and higher social vulnerability) affect the invasion of rural houses by some species of triatomines [i.e. 5,12,18]. Ecological drivers, such as the attraction to artificial lights, have usually concentrated the attention of researchers [12, 18]. Our results suggest that part of the infestation risk associated with a high density of surrounding green vegetation is also tied to vulnerable social conditions of marginalized households inhabiting these landscape types. These results and interpretations are in line with those suggested by Vázquez-Prokopec et al. [17], whereby the expansion of the agricultural frontier brought significant changes in land tenure, land cover and socioeconomic conditions of rural populations in northwestern Argentina. Within large land tracts, mud-and-thatch houses were replaced by “better-built” houses for farm owners and employees prior to complete deforestation [17]. Rural villagers on a subsistence regime (mainly by raising goats and poultry, family agriculture, hunting and gathering) were displaced to the margins of those land tracts [64]. Deforestation and changes in land use were associated with the movement of triatomine vectors and hosts and increased house infestation and host infection risks [65, 66].

The spatial aggregation of domestic infestation and Qom ethnicity qualitatively coincides with the patterns recorded in Areas I [25], Area III [7] and across Pampa del Indio over a decade [22]. The spatial structure uncovered in domestic infestation, Qom ethnicity, suitable walls, surrounding NDVI and presence of peridomestic structures largely supported the inclusion of spatial effects in infestation risk assessments, which showed lower WAIC values. These models identified two main regions with infestation risk not explained by the sociodemographic and environmental variables included in the fixed-effects models. The main region with the largest size and a positive risk of domestic infestation across both intervention periods occurred in Area I [22], where house infestation over the first 3 years postintervention was strongly associated with mud walls, thatched roofs, high refuge availability for triatomines, domestic host abundance and lack of domestic use of insecticides [25]. Area I displayed the highest prevalence of preintervention house infestation (41.4%) and required proportionally more house sprays with pyrethroid insecticide than the rest of the municipality [22]. Household (re)infestation in Area I was closely linked to the occurrence of pyrethroid-resistant *T. infestans* populations [67] and proximity to Castelli, a heavily infested district with high pyrethroid resistance [68, 69]. High contact rates and proximity between households in Area I and Castelli most likely facilitated the passive transport and active dispersal of *T. infestans* to non-infested houses within Pampa del Indio.

The second region with a differential (negative) risk of domestic infestation preintervention was located in Area II, which had the lowest prevalence of house infestation (14.4%) at preintervention across the district [22]. This is partially explained by the fact that the local healthcare system had sprayed some Area II villages with pyrethroids 2 years before the initial community-wide survey and insecticide spraying campaign, whereas in other sections the latest official vector control measures had occurred 6–12 years before [26]. Another distinctive feature of Area II is that the preintervention survey of house infestation comprised a systematic sample (33%) of all houses, unlike the full coverage achieved in other areas. However, postintervention surveys in Area II achieved full coverage and recorded comparatively low degrees of domestic infestation and triatomine infection with *T. cruzi* [22, 24].

Our analysis faced some limitations:

i. The number of missing sociodemographic data determined a smaller dataset with complete data (734). While this represents a considerable subset of the district-wide population, future efforts to compile complete datasets of sociodemographic variables will enable exploring other associations. The estimated credible intervals of the spatial effects and the fixed effects of Qom ethnicity and surrounding NDVI, obtained with the subset of 734 houses, overlapped with those obtained with the subset of 1982 houses (Additional file 2: Text S1, Table S3, and Figure S9). In addition, the missing data could be linked to the high internal mobility rates registered in some sections of Pampa del Indio; hence, data loss might not be completely at random [7, 9].
ii. Domestic infestation assessed by timed manual searches has limited sensitivity to detect low-density infestations, such as those recorded postintervention [70, 71]. Multiple search occasions postintervention largely compensated [22].
iii. Since a considerable percentage of the study houses (15.1%) lacked peridomestic structures, peridomestic infestation was not included in the models although peridomestic infestation is usually associated with domestic infestation and reinfestation processes with *T. infestans* [72]. Recently, peridomestic infestation by *T. infestans* was associated with vegetation cover type around rural houses in northwest Córdoba (Argentina) with higher infestation risk observed in peridomiciles immersed in open shrubland [73].
iv. NDVI has been widely used to measure the density of green vegetation; it may not differentiate between certain vegetation types [14]. During the study period, the land cover of rural Pampa del Indio had a high percentage of forests [30]. The incorporation of vegetation type classification based on field-collected data would add more precision to the environment characterization. Furthermore, climatic conditions can also influence the vegetation characterization, such as temperature and rainfall [74], which also affect the geographical distribution of triatomines [75]. These variables are probably more relevant at larger spatial scales and are less informative at the spatial scale of our study.
v. Unlike other studies that discretized NDVI to facilitate vegetation characterization, we have used NDVI as a continuous variable because its discretization implies a loss of information and statistical power; it increases the probability of false-positive results and may lead to residual confounding [76]. Using a NDVI discretization or a thematic land cover classification would imply adding more variables to regression models, exceeding the limit of the recommended number of variables mainly during the postintervention period [77].
vi. The outcomes of hierarchical Bayesian models may be influenced by the parameters used to create the space triangulation, especially the shortest allowed distance between points and the maximal edge length for the inner domain [49]. Although there is still no formal procedure to specify the triangulation parameters a priori, good practices for its construction are in place [49]. The fact that the ρ estimated by the models was considerably greater than the maximum edge length for the inner domain suggests a minimal influence of the selected triangulation parameters.

### Implications for vector surveillance and control

Our study provides evidence for prioritizing and guiding vector and disease control actions. First, we showed a close link between selected sociodemographic factors and the density of green vegetation surrounding houses. These results broaden the interpretations of domestic infestation risk associated with green vegetation. For domiciliated triatomines with sparse or no sylvatic foci, explanations relying on mechanistic processes linked to sylvatic sources of triatomines may not be appropriate. Further studies would allow clarifying the direct and indirect links between environmental and sociodemographic features and consequently their association with domestic infestation risk by domiciliated triatomines. Second, we showed the importance of integrating spatial, social and environmental aspects for estimating the domestic infestation risk and for the design of surveillance and control actions. Although these aspects had been previously analyzed separately [22], in this study we showed the potential of hierarchical Bayesian models to consider them simultaneously. In this sense, the zones with high infestation risk not explained by socio-environmental variables included in the models are areas in which it is necessary to deepen research to understand which other characteristics may intervene in these domestic infestations. Targeting surveillance and control actions to priority areas would increase the cost-effectiveness of interventions and sustainability of disease control programs.

## Conclusions

Our study showed the need to simultaneously consider social, environmental and spatial aspects in the estimation of domestic infestation risk with *T. infestans* to control the potential dependency across aspects and to improve the understanding of the processes underlying domestic infestation risk. The association between the green vegetation surrounding houses and household social characteristics showed the need to consider non-mechanistic interpretations of the domestic infestation risk associated with the surrounding landscape, mainly in the study of domiciliated triatomines. Vulnerable social conditions adjusted for environmental and spatial characteristics were strongly associated with a higher domestic infestation risk with *T. infestans*. These results reinforce the need to improve housing quality and living conditions to reduce domestic infestation risk with triatomines and improve health. Hierarchical Bayesian models allowed us to integrate social, environmental and spatial effects and identify regions with differential domestic infestation risks not explained by the socio-environmental variables included in the models: this information is key for evidence-based decision-making in the context of vector and disease control.

### Supplementary Information

Supplementary material 1: Dataset S1. House-level infestation data for 734 houses in Pampa del Indio. Dataset S2. House-level infestation data for 1982 houses in Pampa del Indio.

Supplementary material 2: Text S1. Description of the analysis of the subset of 1982 houses. Table S3. Coefficients of fixed and random effects of domestic infestation models in the dataset for 1982 houses. Figure S9. Observed domestic infestation status and posterior median of spatial effects by intervention period in the dataset for 1982 houses.

Supplementary material 3: Figure S1. Spatial distribution of domestic infestation status by intervention period. Figure S2. Spatial distribution of ethnicity by intervention period. Figure S3. Spatial distribution of surrounding NDVI by intervention period. Figure S4. Spatial distribution of suitable walls for triatomines by intervention period. Figure S5. Spatial distribution of the presence of peridomestic structures by intervention period. Figure S6. Spatial distribution of overcrowding by intervention period. Figure S7. Spatial distribution of the number of poultry by intervention period. Figure S8. Spatial distribution of the number of dogs and cats by intervention period.

Supplementary material 4: Table S1. Association of surrounding NDVI with sociodemographic variables. Table S2. Coefficients of fixed and random effects of domestic infestation models using other buffer radii to calculate the surrounding NDVI.

## Data Availability

Data supporting the conclusions of this article are included within the article and its additional files.

## Abbreviations

NDVI: Normalized difference vegetation index
SPDE: Stochastic partial differential equation
WAIC: Watanabe-Akaike information criteria
CI: Credible interval
10M: 10 De Mayo
CT: Campo Los Toros
CO: Colonia Ombú
SV: El Salvaje
CV: Los Ciervos
FB: Fortín Brown
H: La Herradura
LL: La Loma
BV: Las Bravas
CHU: Las Chuñas
RI: Santa Rita
LUG: Santos Lugares
3L: Tres Lagunas
LC: Lote Cuatro
NU: Campo Nuevo
ME: Campo Medina
LA: Cancha Larga
OM: Pampa Ombú
BC: La Barrancosa
PG: Pampa Grande
PC: Pampa Chica
CC: Cuarta Legua Catorce
CD: Cuarta Legua Diecisiete
PV: Pueblo Viejo rural
RN: El Rincón
CQ: Campo Cacique
H2: La Herradura 2
CY: Campo Alemany
CM: Colonia Mixta
LM: Las Muñecas
EP: Ex-Parque
TCZ: Tacuruzal
PIN: Parque industrial
