# Supplementary material for: Domestic (re)infestation risk with the main vector Triatoma infestans increases with surrounding green vegetation and social vulnerability in the Argentine Chaco
Source: Parasit Vectors. 2024 May 27;17:240. doi: 10.1186/s13071-024-06324-3 (PMC11131304; doi:10.1186/s13071-024-06324-3)
Supplement: Supplementary file 2 — Supplementary material 2: Text S1. Description of the analysis of the subset of 1982 houses. Table S3. Coefficients of fixed and random effects of domestic infestation models in the dataset for 1982 houses. Figure S9. Observed domestic infestation status and posterior median of spatial effects by intervention period in the dataset for 1982 houses. [file 13071_2024_6324_MOESM2_ESM.docx]

**Domestic (re)infestation risk with the main vector *Triatoma infestans* increases with surrounding green vegetation and social vulnerability in the Argentine Chaco**

**Additional file 2:** Domestic infestation models in the dataset with 1982 houses

**Text S1.** Only 32.7% (734) of the houses had complete sociodemographic data. Therefore, we also estimated the domestic infestation risks by including 86.9% (1982) of the houses that had complete information for at least ethnicity and surrounding NDVI. In the pre-intervention period, 16.2% (230/1422) of the evaluated houses had a domestic infestation, while in the postintervention period 4.3% (82/1912) of them had a domestic infestation. To estimate the domestic infestation risks we used the same methodology described in the *Domestic infestation model* section of the paper, based on a logistic regression model with a hierarchical Bayesian framework composed by fixed effects and spatial random effects. Table S3 shows the coefficients of the fixed and random effects of the domestic infestation models, in which the 95% credible intervals overlap with those obtained with the subset of 734 houses. While Figure S9 shows the spatial effects by intervention period, in which it is observed that the regions of greater area, which did not include 0 in their 95% credible intervals, overlap with those obtained with the subset of 734 houses.

| **Variable** | **Preintervention** | **Postintervention** |
| --- | --- | --- |
|  |  |  |
| Intercept | -3.12 (-3.92 - -2.47) | -4.13 (-4.98 - -3.45) |
| Qom ethnicity | 1.35 ( 0.83 - 1.88) | 0.24 (-0.42 - 0.92) |
| Surrounding NDVI | 0.23 (-0.09 - 0.55) | 0.56 ( 0.19 - 0.95) |
| Range (ρ) | 6915.68 (4405.37 - 12195.85) | 3547.74 (2140.56 - 6773.11) |
| Standard deviation (σ) | 1.57 ( 1.15 - 2.17) | 1.43 ( 1.13 - 1.83) |

**Table S3.** Median and 95% credible intervals of fixed and random effects of domestic infestation models in the dataset for 1982 houses. Abbreviations: NDVI, normalized difference vegetation index.


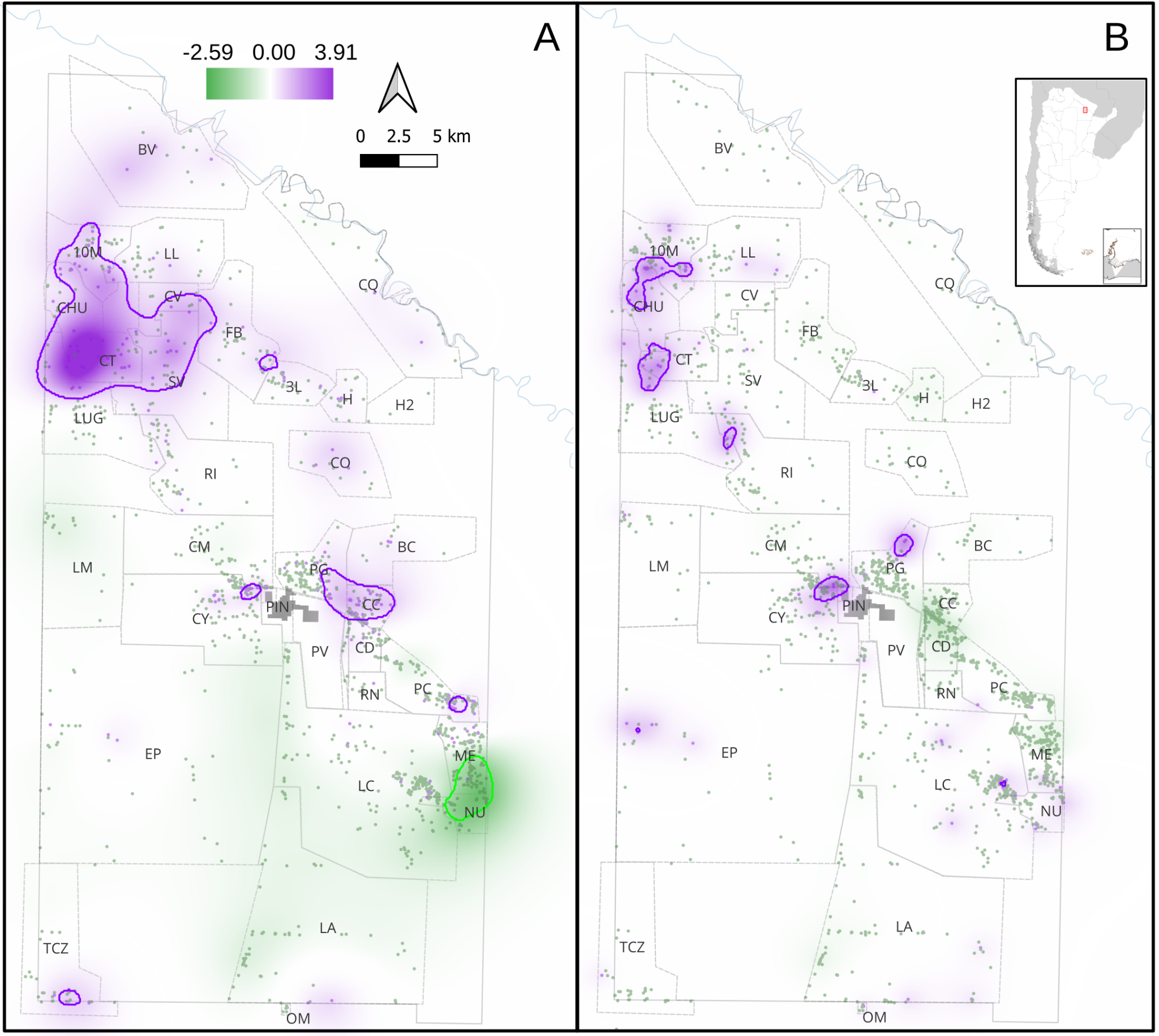


**Figure S9.** Observed domestic infestation status and posterior median of spatial effects by intervention period in the dataset for 1982 houses. A, preintervention and B, postintervention. The regions delimited by violet and green lines correspond to regions where the spatial effect did not include the value 0 in its 95% credible interval; in violet spatial effects greater than 0, in green effects lower than 0. The dots represent houses (violet: infested, green: non-infested). The solid gray lines represent the municipality boundaries, the solid blue line represents the Bermejo River, and the dashed gray lines represent the village boundaries. The gray region represents the urban conglomerate of Pampa del Indio. Village acronyms: 10M, 10 de Mayo; CT, Campo Los Toros; CO, Colonia Ombú; SV, El Salvaje; CV, Los Ciervos; FB, Fortín Brown; H, La Herradura; LL, La Loma; BV, Las Bravas; CHU, Las Chuñas; RI, Santa Rita; LUG, Santos Lugares; 3L, Tres Lagunas; LC, Lote Cuatro; NU, Campo Nuevo; ME, Campo Medina; LA, Cancha Larga; OM, Pampa Ombú; BC, La Barrancosa; PG, Pampa Grande; PC, Pampa Chica; CC, Cuarta Legua Catorce; CD, Cuarta Legua Diecisiete; PV, Pueblo Viejo rural; RN, El Rincón; CQ, Campo Cacique; H2, La Herradura 2; CY, Campo Alemany; CM, Colonia Mixta; LM, Las Muñecas; EP, ex-Parque; TCZ, Tacuruzal; PIN, Parque Industrial.
