# Supplementary material for: Domestic (re)infestation risk with the main vector Triatoma infestans increases with surrounding green vegetation and social vulnerability in the Argentine Chaco
Source: Parasit Vectors. 2024 May 27;17:240. doi: 10.1186/s13071-024-06324-3 (PMC11131304; doi:10.1186/s13071-024-06324-3)
Supplement: Supplementary file 3 — Supplementary material 3: Figure S1. Spatial distribution of domestic infestation status by intervention period. Figure S2. Spatial distribution of ethnicity by intervention period. Figure S3. Spatial distribution of surrounding NDVI by intervention period. Figure S4. Spatial distribution of suitable walls for triatomines by intervention period. Figure S5. Spatial distribution of the presence of peridomestic structures by intervention period. Figure S6. Spatial distribution of overcrowding by intervention period. Figure S7. Spatial distribution of the number of poultry by intervention period. Figure S8. Spatial distribution of the number of dogs and cats by intervention period. [file 13071_2024_6324_MOESM3_ESM.docx]

**Domestic (re)infestation risk with the main vector *Triatoma infestans* increases with surrounding green vegetation and social vulnerability in the Argentine Chaco**

**Additional file 3:** Global spatial analysis of variables


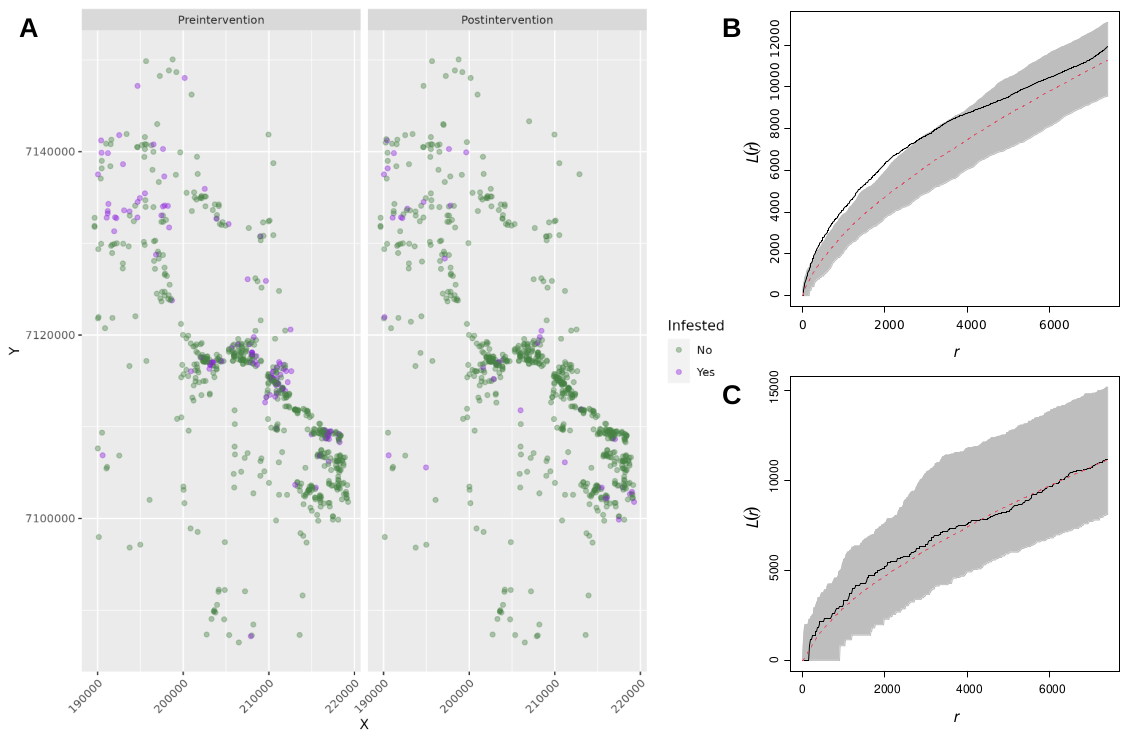


**Figure S1**. Spatial distribution of domestic infestation status by intervention period. Panel A) shows the spatial distribution of domestic infestation status by intervention period; coordinates are shown in meters. Panels B) and C) show the univariate global spatial analysis for domestic infestation in pre and postintervention periods, respectively; the black line indicates the observed Ripley statistic [L(r)], the dashed red line indicates the expected value, and the gray area shows the 95% confidence envelope; the distance (r) is shown in meters.


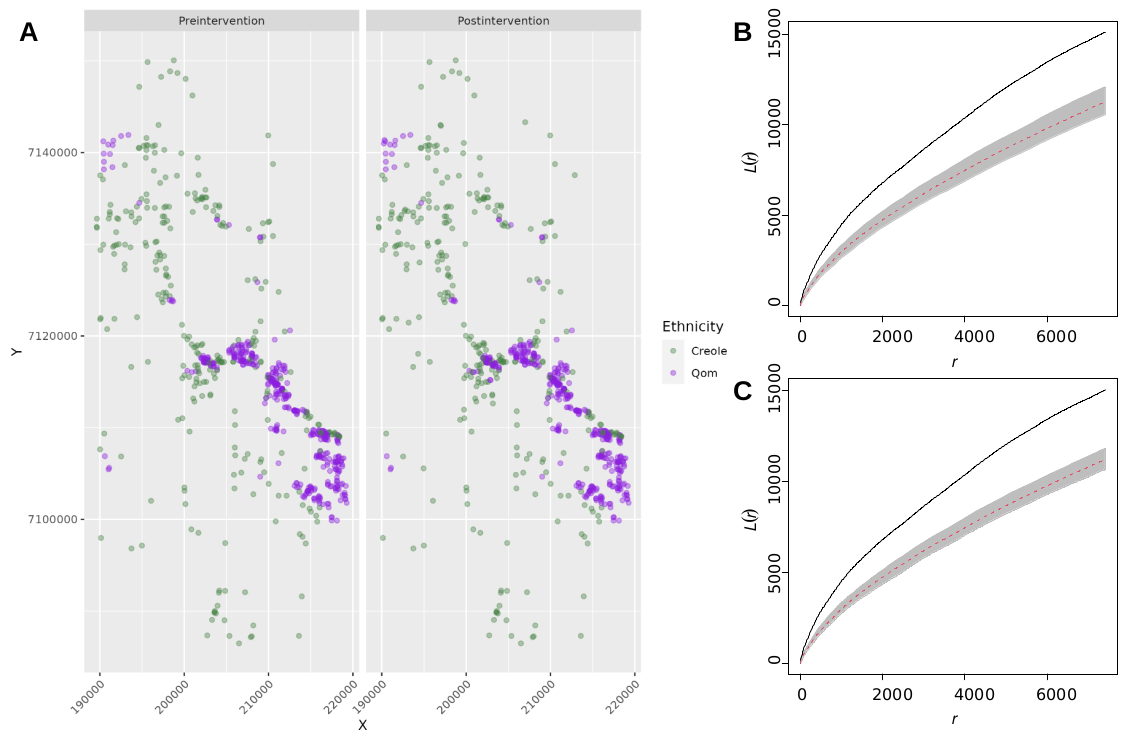


**Figure S2**. Spatial distribution of ethnicity by intervention period. Panel A) shows the spatial distribution of ethnicity by intervention period; coordinates are shown in meters. Panels B) and C) show the univariate global spatial analysis for ethnicity in pre and postintervention periods, respectively; the black line indicates the observed Ripley statistic [L(r)], the dashed red line indicates the expected value, and the gray area shows the 95% confidence envelope; the distance (r) is shown in meters.


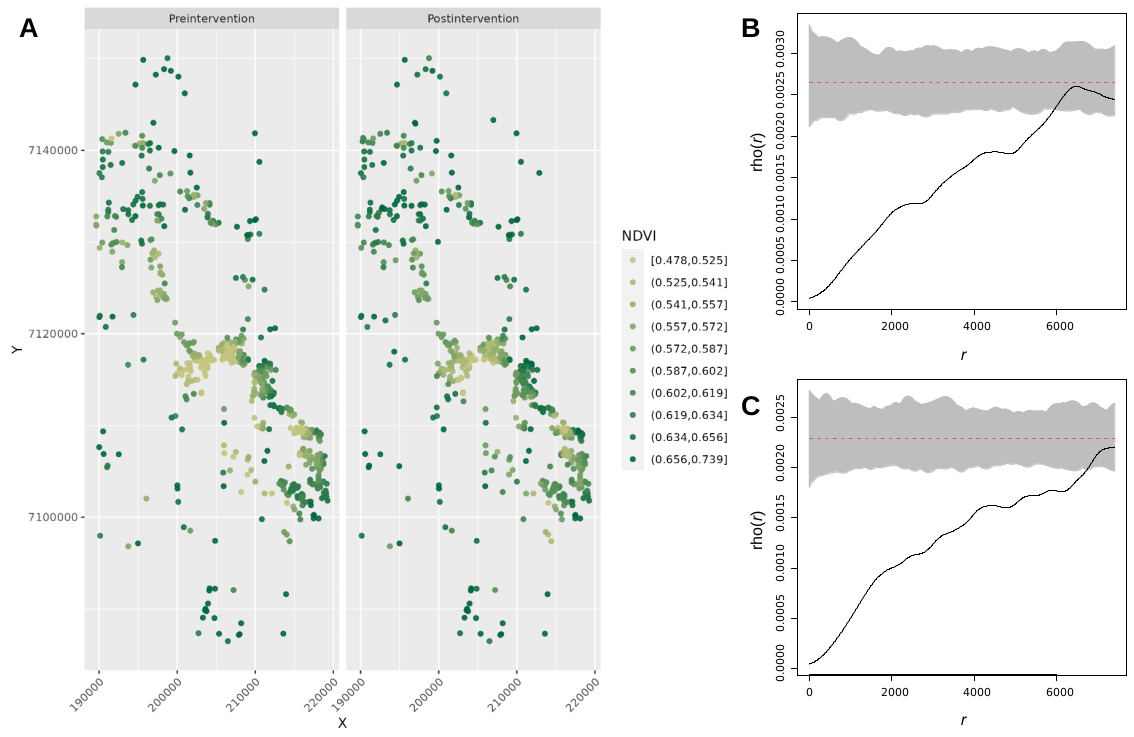


**Figure S3**. Spatial distribution of surrounding NDVI by intervention period. Panel A) shows the spatial distribution of surrounding NDVI by intervention period; coordinates are shown in meters; the surrounding NDVI distribution was classified based on its 10 decentiles. Panels B) and C) show the univariate global spatial analysis for surrounding NDVI in pre and postintervention periods, respectively; the black line indicates the observed mark variogram [rho(r)], the dashed red line indicates the expected value, and the gray area shows the 95% confidence envelope; the distance (r) is shown in meters. Abbreviations: NDVI, normalized difference vegetation index.


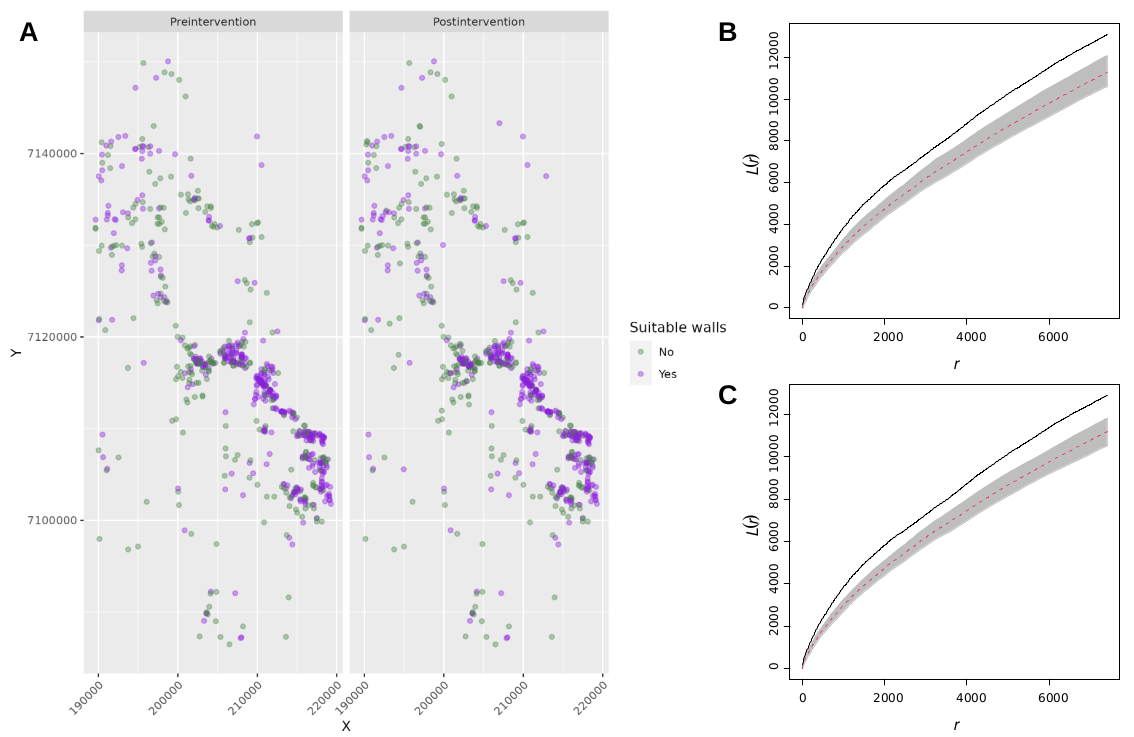


**Figure S4**. Spatial distribution of suitable walls for triatomines by intervention period. Panel A) shows the spatial distribution of suitable walls for triatomines by intervention period; coordinates are shown in meters. Panels B) and C) show the univariate global spatial analysis for suitable walls in pre and postintervention periods, respectively; the black line indicates the observed Ripley statistic [L(r)], the dashed red line indicates the expected value, and the gray area shows the 95% confidence envelope; the distance (r) is shown in meters.


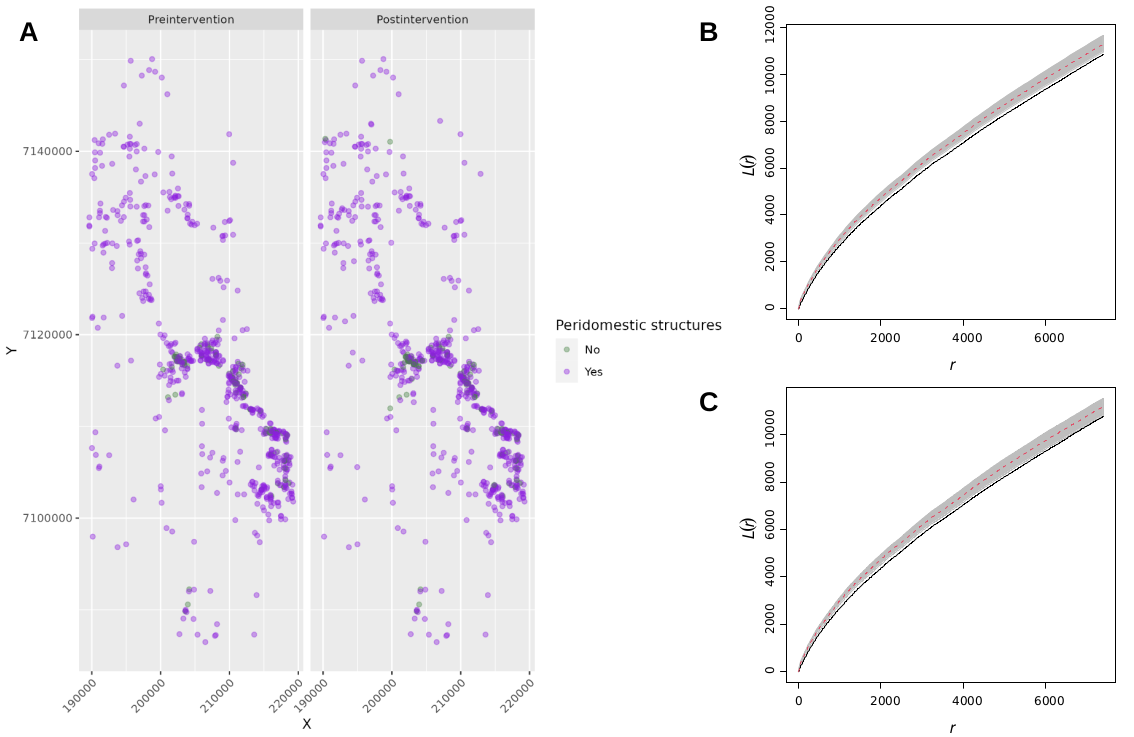


**Figure S5**. Spatial distribution of the presence of peridomestic structures by intervention period. Panel A) shows the spatial distribution of the presence of peridomestic structures by intervention period; coordinates are shown in meters. Panels B) and C) show the univariate global spatial analysis for the presence of peridomestic structures in pre and postintervention periods, respectively; the black line indicates the observed Ripley statistic [L(r)], the dashed red line indicates the expected value, and the gray area shows the 95% confidence envelope; the distance (r) is shown in meters.


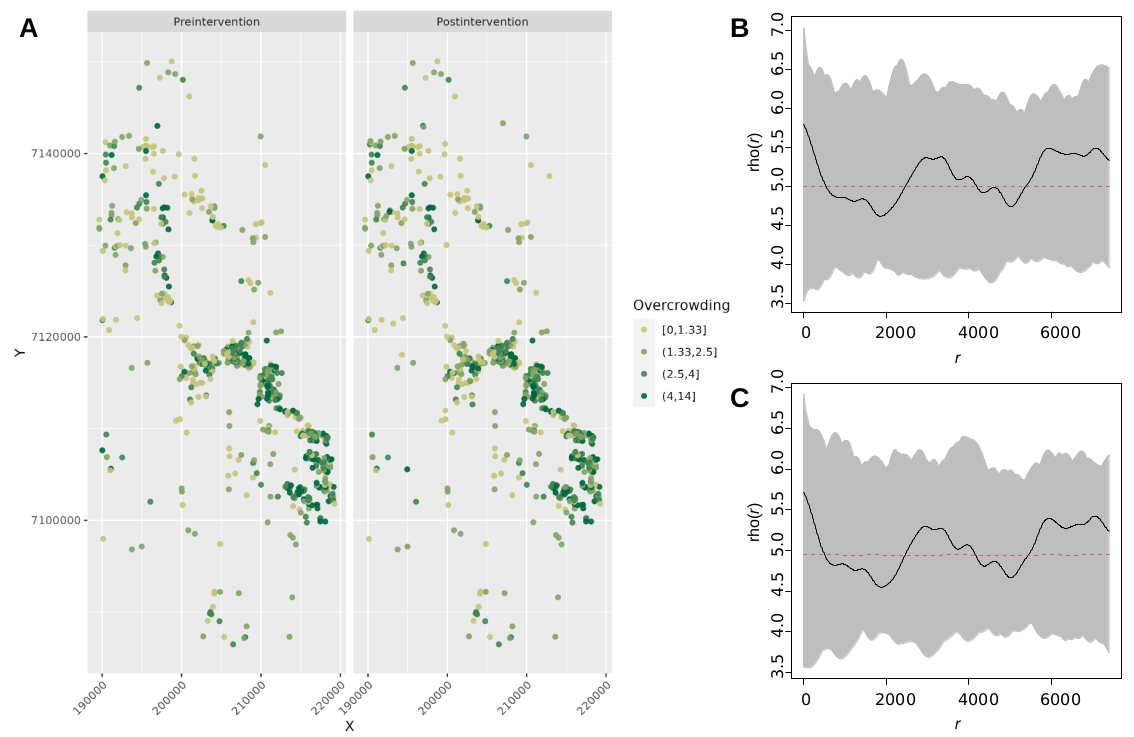


**Figure S6**. Spatial distribution of overcrowding by intervention period. Panel A) shows the spatial distribution of overcrowding by intervention period; coordinates are shown in meters; the overcrowding distribution was classified based on their quartiles. Panels B) and C) show the univariate global spatial analysis for overcrowding in pre and postintervention periods, respectively; the black line indicates the observed mark variogram [rho(r)], the dashed red line indicates the expected value, and the gray area shows the 95% confidence envelope; the distance (r) is shown in meters.


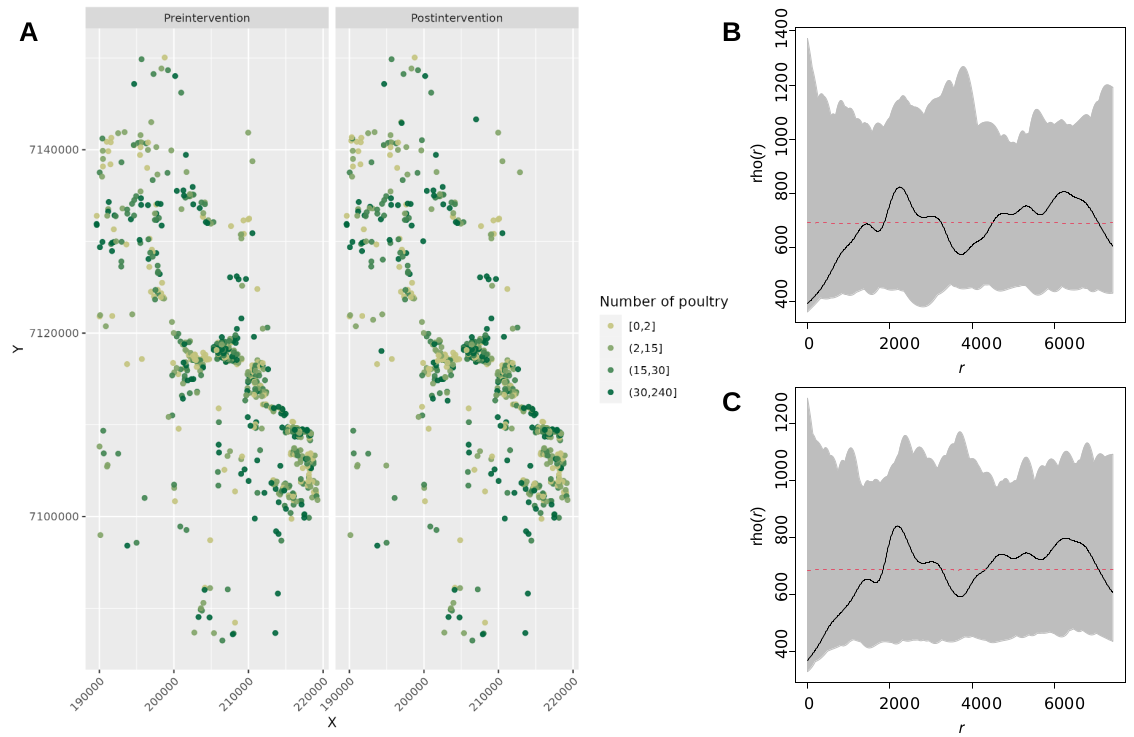


**Figure S7**. Spatial distribution of the number of poultry by intervention period. Panel A) shows the spatial distribution of the number of poultry by intervention period; coordinates are shown in meters; the distribution of the number of poultry was classified based on their quartiles. Panels B) and C) show the univariate global spatial analysis for the number of poultry in pre and postintervention periods, respectively; the black line indicates the observed mark variogram [rho(r)], the dashed red line indicates the expected value, and the gray area shows the 95% confidence envelope; the distance (r) is shown in meters.


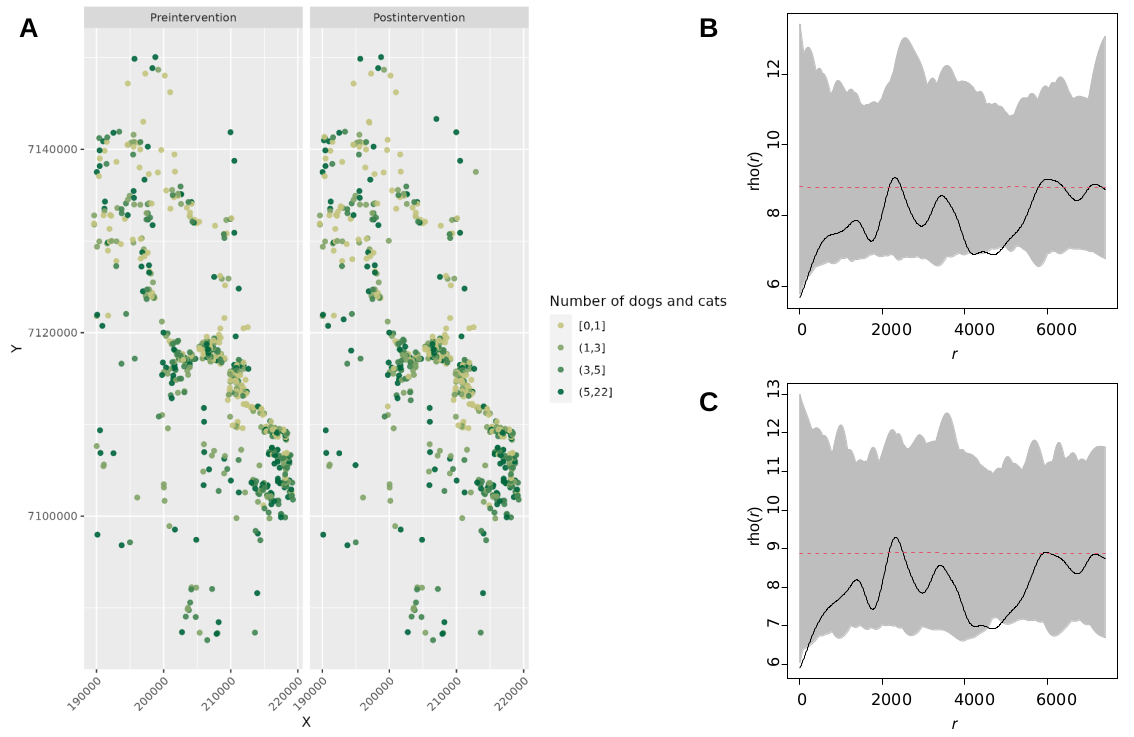


**Figure S8**. Spatial distribution of the number of dogs and cats by intervention period. Panel A) shows the spatial distribution of the number of dogs and cats by intervention period; coordinates are shown in meters; the distribution of the number of dogs and cats was classified based on their quartiles. Panels B) and C) show the univariate global spatial analysis for the number of dogs and cats in pre and postintervention periods, respectively; the black line indicates the observed mark variogram [rho(r)], the dashed red line indicates the expected value, and the gray area shows the 95% confidence envelope; the distance (r) is shown in meters.
